# Investigation of Structural and Optical Properties of Some [1,4]Dithiine-porphyrazine Dyes

**DOI:** 10.3390/molecules27051651

**Published:** 2022-03-02

**Authors:** Ola A. Abu Ali, Hamada H. Abdel-Razik, Matokah Abualnaja, Eman Fayad

**Affiliations:** 1 Department of Chemistry, College of Science, Taif University, P.O. Box 11099, Taif 21944, Saudi Arabia; o.abuali@tu.edu.sa; 2 Chemistry Department, College of Science, Damietta University, New Damietta 34517, Egypt; 3 Department of Chemistry, Faculty of Applied Science, Umm Al-Qura University, Makkah al-Mukarramah 21955, Saudi Arabia; mmabualnaja@uqu.edu.sa; 4 Department of Biotechnology, Faculty of Sciences, Taif University, P.O. Box 11099, Taif 21944, Saudi Arabia

**Keywords:** 1,4-bis(p-tolylamino), 6,7-dichloroanthraquinone, thianone, cyclotetramerization

## Abstract

1,4-Bis(p-tolylamino)-6,7-dichloroanthraquinone **1** when reacted with di(sodiothio)-maleonitrile **2** afforded heterocyclic thianone compound, 5,12-dioxo-5,12-dihydroanthro[2,3-b][1,4]dithiine-2,3-dicarbonitrile 3. Using lithium/pentanol and acetic acid, the dicarbonitrile product **3** was cyclotetramerized, yielding the matching tetra 5,12-dioxo-5,12-dihydroanthro[2,3-b][1,4]dithiine-porphyrazine dye compound (2H-Pz) **4a**. The dicarbonitrile molecule was a ring-shaped metallic product utilizing metallic salt and quinoline, yielding the corresponding tetra 5,12-dioxo-5,12-dihydroanthro[2,3-b][1,4]dithiine-porphyrazinato-metal II dyes (M-Pz), M = Zn, Co, or Ni **4b**–**d**. The produced compounds’ elemental analysis investigation, Infrared, and nuclear magnetic resonance spectrum information accord with the structures attributed to them. The cyclotetramerization and complexation reactions are ensured by the molecular weight and metal load of the produced products. The inclusion of electron-donating groups resulted in a lower optical band gap of the produced dye sensitizers, with “push–pull” promotion of about 1.55 eV. The prepared substituted porphyrazines reveal high absorption in the UV–VIS region, which could be of potential value as a building block for novel electronic and optical materials as well as a sensor for technology. This is considered for improving solar cell absorption. The absorption bands of the synthesized porphyrazine dyes extend beyond 800 nm, so these dyes could be useful in various optoelectronic applications.

## 1. Introduction

In the broadest sense, optical substances change or control electromagnetic radiation in the ultraviolet (UV), visible, or infrared (IR) spectrum areas. These dyes are made into optical elements including lenses, reflections, screens, prisms, polarizers, sensors, and stimulators. Atoms and their electrical structures in the material interact with electromagnetic radiation (photons) at the microscopic scale to establish macroscopic optical properties. The external electric and magnetic fields applied to the material determine these optical properties. Optical materials can be made out of a wide variety of organic or inorganic materials [1]. Tetra-(1,4-dithiin)porphyrazine is one of the phthalocyanine derivatives having two sulfur atoms at equivalent 1,4-positions of the phthalocyanine benzene units [2]. Zinc, aluminium, and other metal porphyrazine complexes have anticancer characteristics and are used in photodynamic treatment [3]. In a multi-step reaction sequence, novel tetra-(1,4-dithiin) metal-free (H2-Pz) and metallo-porphyrazines (M-Pz, M = Mg, Fe) carrying peripheral tetra propyl-bromine were synthesized from 2,3-dicyano-5-propyl-bromine-1,4-dithiin and disodiomaleonitrile [4]. It was discovered that a new catalyst, iron (II) tetra (1,4-dithin)porphyrazine, can be used to oxygenate the breakdown of organic contaminants in aqueous solutions [5]. The peripherally peralkynylated phthalocyanine or naphthalocyanine analogues, namely tetra pyrazinoporphyrazines and tetra-6,7-quinoxalinoporphyrazines, are generated via base-induced cyclotetramerization of aromatic dinitriles with magnesium butoxide in refluxing butanol [6]. Iron (II)-tetramethyl-tetra (1,4-dithiin)porphyrazine was synthesized, and its photocatalytic characteristics were investigated [7]. The synthesis of metal-free and magnesium tetra-ester tetra(1,4-dithiin)porphyrazine was accomplished [8].

Zinc-tetra (1,4-dithiin)porphyrazine containing peripheral tetra propyl-bromine was synthesized, and its photocatalytic capabilities were described [9]. The photo-catalytic activity of peripheral substituents of cobalt thioporphyrazines has been described [10,11]. The photophysical and electrochemical properties of two porphyrin-chromophores have been explored [12]. The photophysical characteristics of three carbazole-fused zinc metallic porphyrin sensitizers were examined [13]. Porphyrazines are made by cyclizing maleonitrile with a magnesium template. Starting with the appropriate unsaturated dicarbonitrile derivative, metallic-porphyrazines were produced [14]. Compounds with more electrons and with low electron portions joined by a linkage have successfully been applied as rising alternatives [15,16,17].

The “push–pull” architecture of these “donor–acceptor” molecules facilitates the isolation of holes and electrons, reducing recombination. By cyclotetramerizing 1,2-bis(4-tert-butylphenylthio)maleonitrile in the presence of magnesium butanolate, magnesium porphyrazinate with eight 4-tert-butylphenylthio-groups on the peripheral locations has been produced [18,19]. Tetra(1,2,5-thiadiazolo)porphyrazine complexes with yttrium(III) and lutetium(III) were prepared, spectrally analyzed, and studied using DFT [20,21]. The electronic and geometric structures of porphyrazine (Pz) and tetrakis(1,2,5-thiadiazole)porphyrazine (TTDPz) metal complexes were studied [22]. There have been reports of organic/metal photosensitizers for dye-sensitized solar cells [23]. In this study, we aimed to create new porphyrazines that could be used as a key component for new electronic and optical compounds, as well as a technology sensor.

## 2. Results and Discussion

1,4-Bis(p-tolylamino)-6,7-dichloroanthraquinone **1** when condensed with di(sodiothio)maleonitrile **2** yielded di-carbonitrile derivative **3**. Using lithium/pentanol and acetic acid, the dicarbonitrile product was cyclotetramerized, yielding tetra 5,12-dioxo-5,12-dihydroanthro[2,3-b][1,4]dithiine-porphyrazine dye (2H-Pz) **4a**. Using metal salt and quinoline, the dicarbonitrile molecule was also cyclotetramerized, yielding the matching tetra 5,12-dioxo-5,12-dihydroanthro[2,3-b][1,4]dithiine-porphyrazinato-metal II dye (M-Pz), M = Zn **4b**, Co **4c**, or Ni **4d**.

**Scheme 1:**
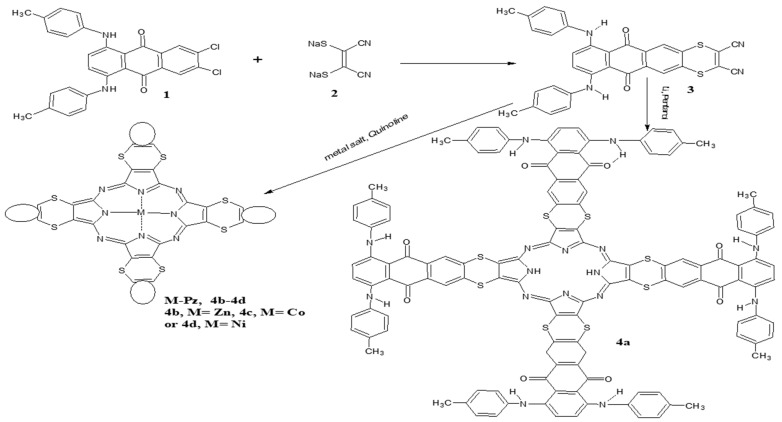
Synthetic reaction rout of di-carbonitrile derivative **3** and porphyrazines **4a**–**4d**.

The number of carbon atoms in the prepared dye structure reflects the envisioned structures presented in Scheme 1. Additionally, the metal concentration of the produced porphyrazines (see experimental) is comparable to that anticipated for the dye’s compositions predicted. The metal concentration (see experimental) and molecular mass of the generated dyes also show that the cyclo-tetramerization and chelation reactions are effective [24].

### 2.1. Infrared Spectra

**Figure 1:**
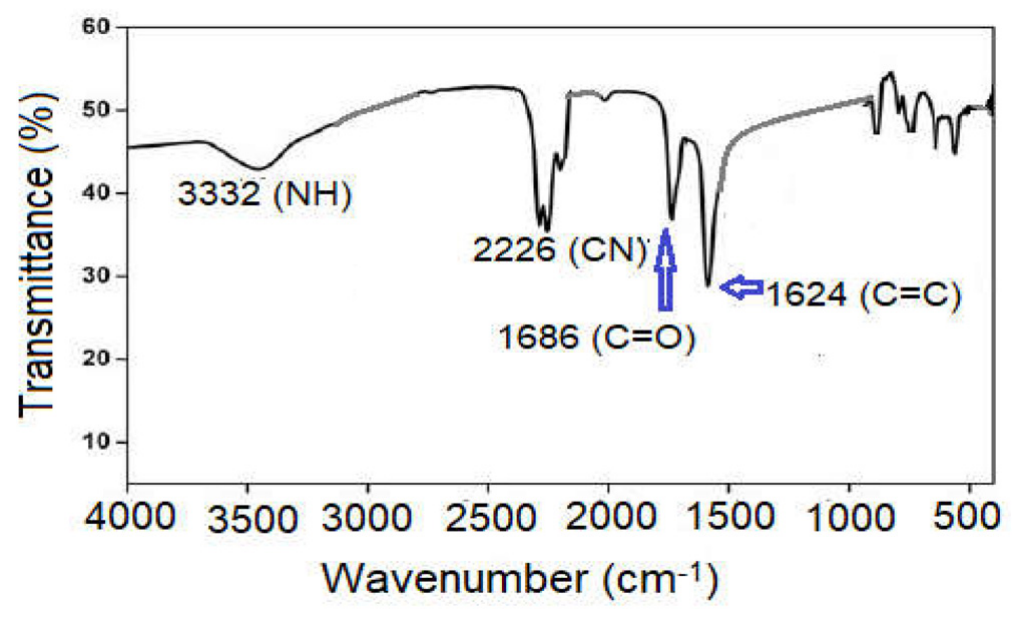
FT-IR spectra of dicarbonitrile **3**.

**Figure 2:**
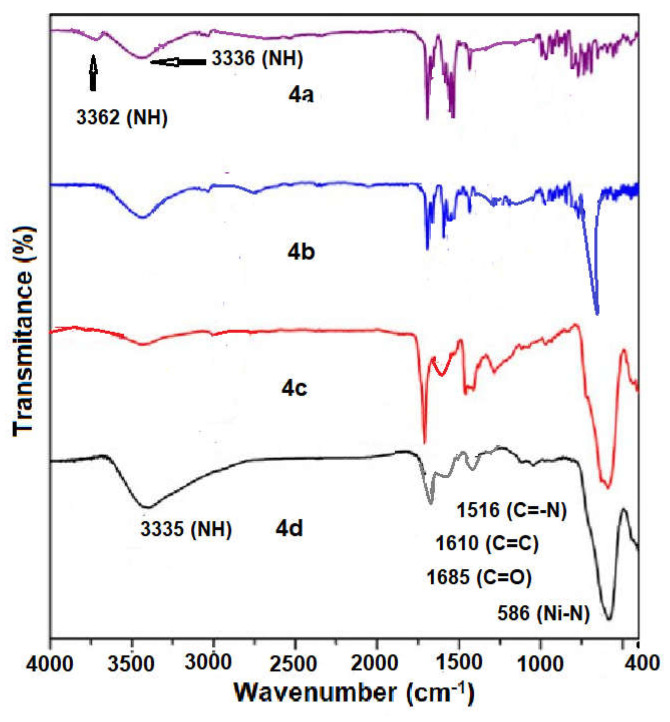
FT-IR spectra of porphyrazines **4a**–**4d**.

Prominent bands at 1624, 3332, 1686, and 2226 cm^−1^ are ascribed to the C=C, NH, C=O, and CN groups throughout the IR spectral information of component **3** (Figure 1). The stretching vibration of the C=N bond is related to the wide peak provided in the infrared investigation of product **4a** at 1522 cm^−1^. The absorption band of the C=N oscillation by Zn-porphyrazine dye **4b**, Co-porphyrazine dye **4c**, and Ni-porphyrazine dye **4d** at 1508, 1512, and 1516 cm^−1^ (see experimental) is roughly 6–14 cm^−1^ lower than that of the porphyrazine derivative **4a**, which depicts the engagement of a nitrogen atom of azomethine moiety with metallic salt ions in combinations. Additionally, a large peak appears at 3362 cm^−1^, which is explained by the oscillation of the N-H link stretching in the component **4a**. Since this oscillation of the N-H link is not found in that observed for dyes **4b**–**4d**, the NH unit is included in chelation process (Figure 2).

### 2.2. Measurements of NMR and Molecular Mass

**Figure 3:**
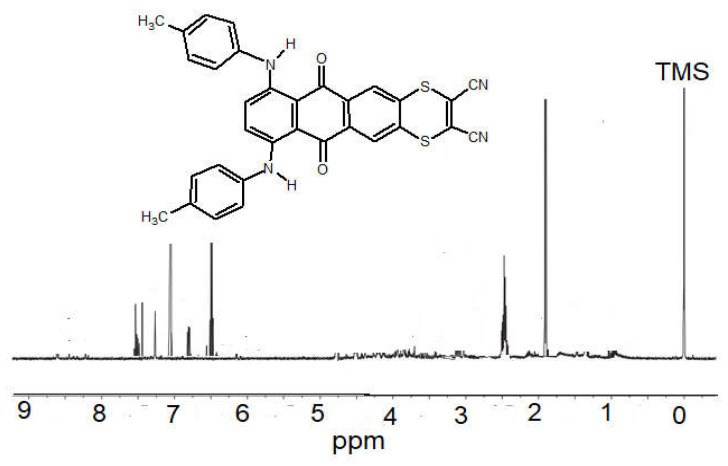
^1^H-NMR spectra of dicarbonitrile **3**.

**Figure 4:**
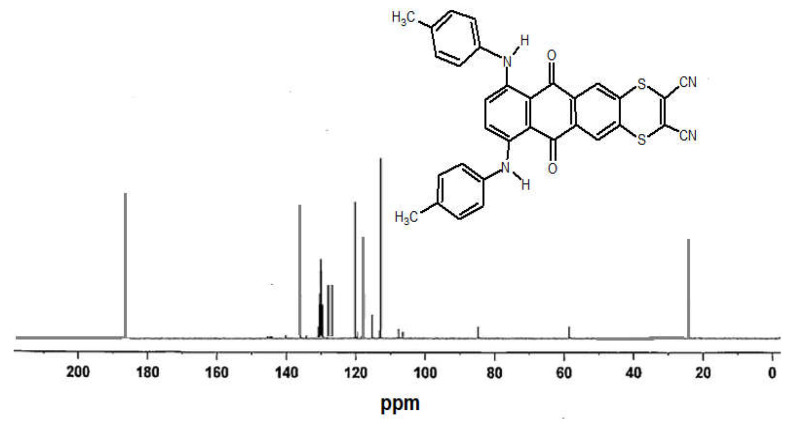
^13^C-NMR spectra of dicarbonitril **3**.

According to the structure of compound **3**, the ^1^H-NMR spectrum shows a band at 6.28–6.85 ppm able to assign anthraquinone ring and benzene ring. Bands at 2.46 and 1.8 ppm belonging to CH_3_ and NH moieties are shown (Figure 3). The ^13^C-NMR spectrum identified bands at 24.4, 117.3, 123.5, 124.5, 127.7, 128.4, 128.8, 130.1, 138.0, and 187.1 ppm corresponding to CH_3_, CN, C=C (thiin ring), C=C (anthraquinone ring, benzene ring), and C=O, respectively (Figure 4). The assigned formulation is compatible with the elemental analytical results of new product **3** (see experimental, Scheme 1).

**Figure 5:**
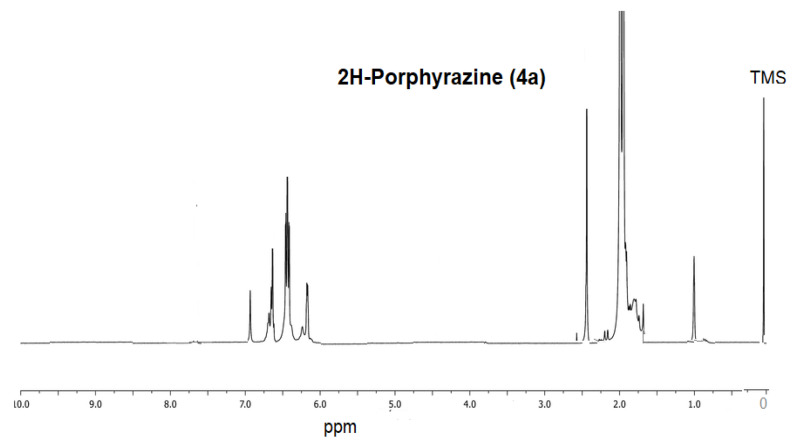
^1^H-NMR spectra of porphyrazine dye **4a**.

**Figure 6:**
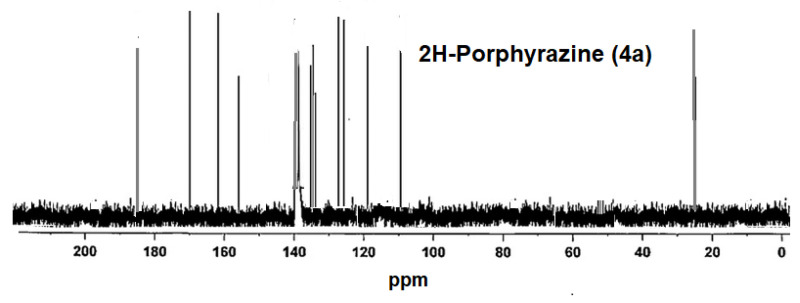
^13^C-NMR spectra of porphyrazine dye **4a**.

**Figure 7:**
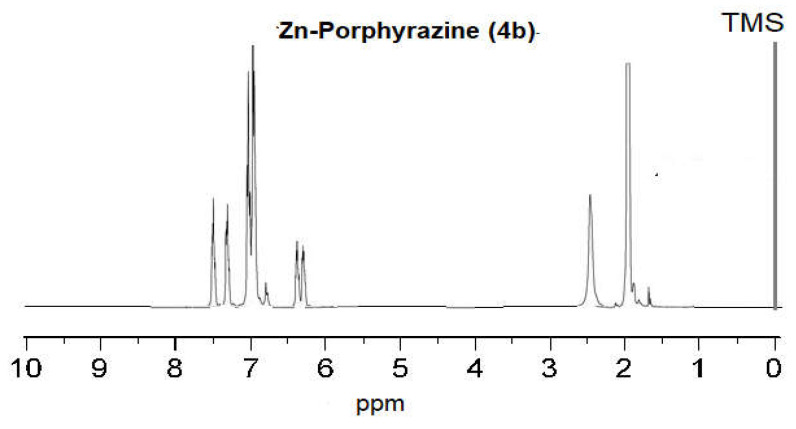
^1^H-NMR spectra of porphyrazine dye **4b**.

**Figure 8:**
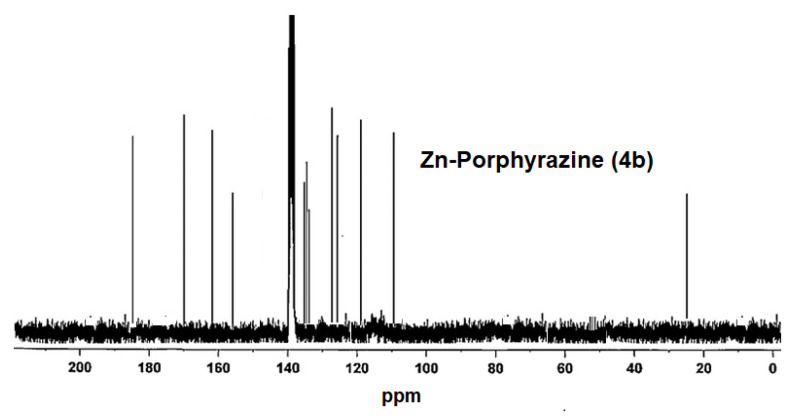
^13^C-NMR spectra of porphyrazine dye **4b**.

**Figure 9:**
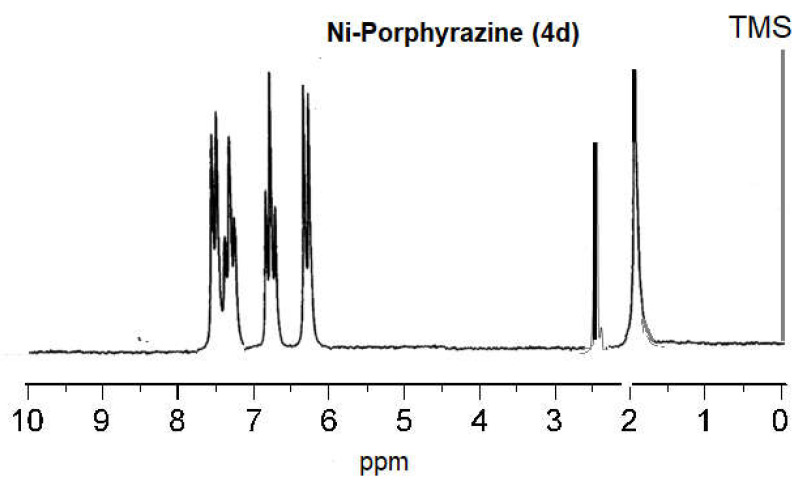
^1^H-NMR spectra of porphyrazine dye **4d**.

**Figure 10:**
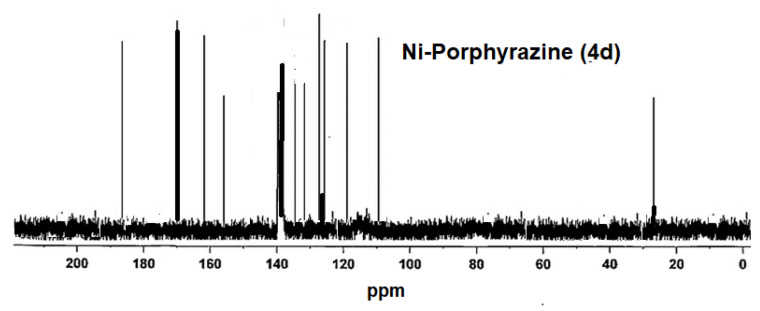
^13^C-NMR spectra of porphyrazine dye **4d**.

^1^H-NMR spectrum of **4a** (Figure 5) show two signals at 1.95 ppm (tolyl-NH) and 0.94 ppm (pyrrole moiety-NH) related to NH protons demonstrating the construction of a tetra-dentate ligand with an unsymmetrical structure. The ^13^C-NMR spectra of product **4a** (Figure 6) revealed a minor peak at 108.8 ppm, which can be attributed to the C=N group, and large bands at 24.8, 119.6, 138.6, 121.8, 134.9, 130.8, 137.0 160.4, and 188.7 ppm, confirming the porphyrazine’s structure. The ^1^H-NMR spectrum of **4b** (Figure 7) shows a signal at 1.95 ppm attributed to tolyl-NH. The absence of a signal in the spectra of **4b** related to the pyrrole moiety-NH ensures metal bonding with the pyrrole moiety-N. Figure 8 shows the ^13^C-NMR spectra of product **4b**, which indicated a minor band at 108.8 ppm that could be assigned to the CN group, as well as big bands at 23.8, 120.6, 137.2, 122.6, 135.2, 130.3, 137.26, 162.4, and 188.6 ppm that confirmed the structure of metal-porphyrazine. The compound **4c** is paramagnetic. The absence of a signal in the nmr spectrum of **4d** related to the pyrrole moiety-NH ensures metal bonding with the pyrrole moiety-N (Figure 9). ^13^CNMR spectrum of Ni-Porphyrazine complex **4d** (Figure 10) is in contestant with its expected structure.

### 2.3. Ultraviolet-Visible Spectra

**Figure 11:**
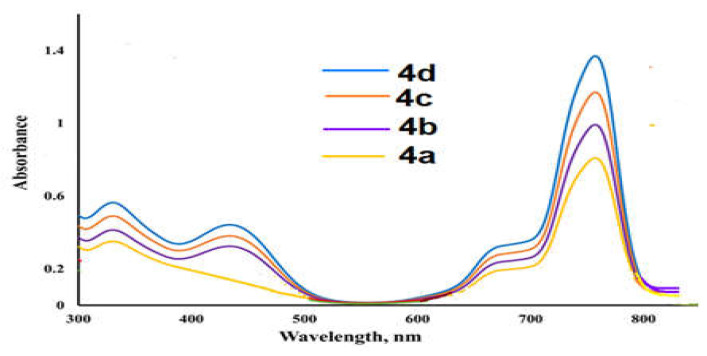
Ultraviolet-visible spectra of the porphyrazines **4a**–**4d**.

A significant soret band at 338 nm may be shown in ultraviolet-visible spectra of **4a** (Figure 11). The energy gap of both the high filled orbital and the low empty orbital enables the π-π* shifts in compound **4a** that are illustrated at 663 and 752 nm. These bands closely resemble the characteristic bands mentioned in the literature [25]. In the characteristic spectroscopic analysis of the metallic contained combinations, **4b**–**d** in ethanol, the n-π* and/or d-π* transformations in the fused pyrrolo (1,4-dithiin) ring structure are conspicuous at larger B-peak (broad bands about 337 nm and narrow peaks at 446 nm) [26,27]. There are two distinct powerful fewer-energy Q-peaks (about 668 and 762 nm) that are highly developed. The transformation from 1s to **4d** is commonly linked to the shoulder band [28]. At shorter wavelengths, interaction of the metal atom with binding modes leads to flip transformations, according to early theoretical studies [29,30]. All metal (1,4-dithiin)porphyrazines have peaks in the region of 436–445 nm beneath the Q-band. These states are caused by ligand metal exchange coupling and d-d excitations. The visible spectra of several metal (1,4-dithiin)porphyrazines varied by a small amount.

Transformation of charge from the metallic atom to the binder has been postulated to influence both the Q and B bands [31]. Metal complexes’ absorption bands clearly extend beyond 800 nm. As a function, these dyes could be beneficial in applications requiring near-infrared absorption, such as optical information storage and safety photocopying. Both of the Soret and Q peaks could be understood using the Gouterman four orbital approach, which emerged from π-π* transitions [32,33,34]. When compared to Co and Ni dyes, the transitions in Zinc dye are broader and moved toward longer wavelengths. The weaker signal at the reduced (elevated) part of the Q zone corresponds to the lowest charge carrier transformation for each dye. For Zn dye, it appears at 2.23 eV (663 nm), whereas for metal free and metal dyes, it occurs at 1.80–2.08 eV (665–672 nm). These energies represent the compounds’ lowest limitations. As a result, the HOMO-LUMO separation of Co and Ni dyes is 0.25 eV fewer than that appeared in Zn dyes. This really is good for sunlight optical absorption since it closes the distance to the solitary cell’s optimal (about 1.1–1.4 eV) [35]. The optical band (Eg) was calculated from the last band edge at the highest frequency in UV-VIS absorption spectra (Figure 9). The last edge wavelength is at 800 nm and the Eg was calculated using the equation, Eg = 1240/λ = 1240/800 = 1.55. In all dyes, it primarily affects the nitrogen atoms of the chromophore, as well as the carbon atoms and anthra unit-accepting moieties. This verifies previous observations that such LUMO in Zn chromic dye is primarily located in the chromic dye canter as just a π*-orbital [36,37]. While the HOMO and LUMO of Zn dyes are situated in the same portion of the structure, notably the porphyrin circle and to a lesser degree the recipient group, there is a large spatial difference of the pair in Co and Ni dyes. The LUMO is part of the acceptor moiety, while the HOMO is part of the donor moiety, with some of them intersecting largely inside the chromophore. When electron-hole pairs in Zn-dye is separated spatially during solar irradiation, the recombination rate is reduced and the output current is increased.

## 3. Materials and Methods

### 3.1. Characterization

All chemicals and solvents were purchased from commercial suppliers and used without purification. N,N-Dimethylformamide (anhydrous, 99.8%), Ethyl alcohol (96.0–97.2%), 1-Pentanol (≥99%), Quinoline (98%), Petroleum ether (AR, bp 40–60 °C), Acetone (≥99.5%), Lithium metal (99%), Zinc chloride (≥98%), Cobalt(II) chloride (97%), and Nickel(II) chloride (98%) were purchased from Sigma-Aldrich (St. Louis, MO, USA). 1,4-Bis(p-tolylamino)-6,7-dichloroanthraquinone {Merck}, and Di (sodiothio) maleonitrile {TCI (Shanghai) Development Co., Ltd., Shanghai, China} were used. Melting points (uncorrected) were determined in open capillaries on the Electrothermal melting point apparatus (Electrothermal Engineering Ltd., Rochford, UK). Spectral measurements were taken with Shimadzu 8101 M Fourier-transform infrared spectrometer. The spectrometer Uni-cam UV-Vis was used to obtain ultraviolet spectra. Utilizing tetramethyl silane as standard procedures, a Varian VXR 400S NMR instrument operated at 400 MHz (^1^H-NMR) and 100 MHz (^13^C-NMR) was used, and NMR spectra were recorded in deuterio-chloroform. The metal content was determined using a Perkin Elmer Analyzer 300, AAS spectrometer. The elemental assessments were obtained utilizing PerkinElmer 2400 CHN. For monitoring the progress of a reaction, thin layer chromatography (TLC) was performed on pre-coated Merck (Darmstadt, Germany) silica gel 60F-254 plates using petroleum ether/acetone (10:5 *v*/*v*) as solvent system.

### 3.2. Synthesis of Dicarbonitrile Derivative ***3***

1,4-Bis(p-tolylamino)-6,7-dichloroanthraquinone (0.01 mol) **1** and (0.01 mol) di (sodiothio) maleonitrile **2** were heated at 80 °C for 8 h in the existence of dimethylformamide (50 mL). The product became brown at the finish of the reaction course period. It was filtered while heated, then deposited by an iced water, rinsed several times with water, left to dry, and crystallized from ethyl alcohol. A total of 88% product (yellowish brown crystallites); MP: 186 °C. Infrared spectra IR (KBr): cm^−1^, 1624 (C=C), 1686 (C=O), 3332 (NH) and 2226 (CN); ^1^H-NMR (CDCl_3_): 1.8 ppm (aromatic C-NH), 2.46 ppm (CH3), 6.28–7.60 ppm (aromatic H). ^13^C-NMR (CDCl_3_): 24.4, 117.3, 119.0, 127.7, 128.4, 130.1, 138.0, and 187.10 ppm. Analysis Calculated for C_32_H_20_N_4_O_2_S_2_ (Mol. Wt.: 556.66) requires: C (69.04%), H (3.62%), N (10.06%). Found: C (69.13%), H (3.68%), N (10.11%).

### 3.3. Synthesis of the Dye Porphyrazine Derivative (2H-Pz) ***4a***

A refluxing solution of **3** (0.5 mmol) in pentanol (100 mL) was mixed with lithium metal (20 mg, 2.8 mmol). The solution was heated for 16 h at reflux. After cooling, it was filtered while still hot, precipitated by an iced water, rinsed many times with H_2_O, dried, and purified to crystals from ethanol. A total of 90% yield (brown crystallites). MP: 212 °C. IR [KBr]: cm^−1^, 1522 (C=N), 1614 (C=C), 1690 (C=O), 3362 (NH), 3336 (NH). UV-Vis λmax(CH_2_Cl_2_)/nm): 338, 663, 752. ^1^H-NMR (CDCl_3_) δ = 6.36–6.97 ppm (aromatic H), 0.94, 1.95 ppm (s, NH), 2.49 ppm (CH_3_). ^13^C-NMR spectrum revealed a small band at 24.8, 108.8 ppm able to assign CH_3_ and C=N groups. Big bands at 119.6, 138.6, 121.8, 134.9, 130.8, 137.0 160.4, and 188.7 ppm. Analytical calculated for the expected porphyrazine C_128_H_84_N_16_O_8_S_8_, Mol. Wt.: 2230.66, requires: C (68.92%), H (3.80%), and N (11.50%). Found: C (68.97%), H (3.86%), and N (10.11%).

### 3.4. Synthesis of the Dye Porphyrazinato-Metal II Derivatives (M-Pz) ***4b**–**4d***

The appropriate metal complex was formed by heating the dinitrile monomer **3** (0.5 mmol) in quinoline (100 mL) at 200 °C for 16 h with 0.75 mmol of metallic salt (Zinc chloride, Cobalt chloride, or Nickel chloride). After the material was dissolved in acetone, the mis metal was recovered and extracted from the solution. It was purified while still heated, deposited with crushed ice, rinsed many times with distilled water, left to dry, and crystallized from ethyl alcohol. Vacuum drying was performed overnight on the product.

**4b**: 93% yield (deep brown crystals), MP: 208 °C. IR (KBr): cm^−1^, 1508, (C=N), 3330 (NH), 1611 (C=C), 1686 (C=O). UV-Vis °max (CH_2_Cl_2_)/nm): 342, 452 (shoulder), 672 and 765. ^1^H-NMR (CDCl_3_) δ = 6.32–7.54 ppm (aromatic H), 1.98 ppm (s, NH), 2.44 ppm (CH_3_). ^13^C-NMR spectrum revealed a small band at 108.8 ppm able to assign C=N group and big bands at 23.8, 120.6, 137.2, 122.6, 135.2, 130.3, 137.26, 162.4, and 188.6 ppm. Analysis calculated for the expected porphyrazine C_128_H_82_N_16_O_8_S_8_Zn (Mol. Wt.: 2294.03) requires: C (67.02%), H (3.60%), N (9.77%), and Zn (2.85%). Found: C (67.11%), H (3.67%), N (9.82%), and Zn (2.87%).

**4c**: 89% yield (Dark-green crystals), MP: 205 °C. IR [KBr]: cm^−1^, 1512, (C=N), 3326 (NH), 1607 (C=C), 1688 (C=O). UV-Vis ºmax (CH_2_Cl_2_)/nm): 337, 446 (shoulder), 668 and 762. Analysis calculated for the expected porphyrazine C_128_H_82_N_16_O_8_S_8_Co (Mol. Wt.: 2287.58) requires: C (67.21%), H (3.61%), N (9.80%), and Co (2.58%). Found: C (67.28%), H (3.66%), N (9.87%), and Co (2.65%).

**4d**: 91% yield (brown crystals), MP: 203 °C. IR [KBr]: cm^−1^, 1516, (C=N), 3335 (NH), 1610 (C=C), 1685 (C=O). UV-Vis °max (CH_2_Cl_2_)/nm): 334, 440 (shoulder), 665 and 759. ^1^H-NMR (CDCl_3_) δ = 6.30–7.57 ppm (aromatic H), 1.95 ppm (s, NH), 2.34 ppm (CH_3_). ^13^C-NMR spectrum revealed a small band at 108.8 ppm able to assign C=N group and big bands at 24.0, 120.3, 138.2, 121.4, 134.3, 131.3, 138.0 161.7, and 185.9 ppm. Analysis calculated for the expected porphyrazine C_128_H_82_N_16_O_8_S_8_Ni (Mol. Wt.: 2287.34) requires: C (67.21%), H (3.61%), N (9.80%), and Ni (2.57%). Found: C (67.28%), H (3.68%), N (9.88%), and Ni (2.66%).

## 4. Conclusions

Tetra free metal- and [1,4]dithiine-porphyrazinato-metal II dye derivatives light sensitizers were produced. The metal concentration and molecular mass of the generated dyes show that the cyclo-tetramerization and chelation reactions are effective. The prepared substituted porphyrazines reveal high absorption in the UV–VIS region, which could be of potential value as a building block for novel electronic and optical materials as well as a sensor for technology. The presence of electron-donating amine groups reduced the optical band gap of organometallic sensitizers with a “push–pull” structure by about 1.55 eV.

## Data Availability

The data presented in this study are available on request from the corresponding author.
